# Pachyméningite et syndrome de Gougerot Sjörgren

**DOI:** 10.11604/pamj.2015.22.88.7840

**Published:** 2015-10-01

**Authors:** Haykel Abdelhedi, Naziha Khammassi

**Affiliations:** 1 Faculté de Médecine de Tunis, Service de Médecine Interne, Hôpital Razi, 2010 La Manouba,Tunisie

**Keywords:** Sydrome de Gougerot Sjörgren, pachyméningite, manifestations neurologiques, Sjörgren syndrome, pachymeningitis, neurological manifestations

## Image en medicine

Les manifestations neurologiques centrales du syndrome de Gougerot-Sjögren primitif (SGS) sont très polymorphes, parmi lesquelles les pachyméningites représentent l'une des manifestations les plus rares, elles sont exceptionnellement révélatrices. Patient âgé de 40 ans présente depuis 7 ans des céphalées fronto-orbitaires gauches pulsatiles avec sono-photophobie. Depuis un an, les céphalées sont devenues plus intenses associées à une sécheresse oculaire et buccale. Une IRM cérébrale a objectivé une prise de contraste intense et épaissie des espaces méningés sous et sus-tentoriels réalisant l'aspect d'une pachyméningite diffuse. A l'examen clinique il n'y avait pas d'aphtose bipolaire ni d’érythème noueux ni de pseudo-folliculites. L'examen de la sphère ORL était normal. La recherche de bacille de Koch dans les crachats ainsi que les sérologies syphilitique et de la maladie de Lyme étaient négatives. Le bilan phosphocalcique et le dosage de l'enzyme de conversion de l'angiotensine étaient normaux ainsi que la radiographie thoracique et la spirométrie. Le bilan immunologique (anticorps antinucléaires, ANCA) était négatif et l’étude du LCR était sans anomalies. Le test de Shirmer était pathologique et la biopsie des glandes salivaires était en faveur d'une sialadinite stade IV de Chisholm confirmant le diagnostic du SGS. Les causes des pachyméningites sont très diverses: infectieuses, inflammatoires, médicamenteuses, néoplasiques ou idiopathiques. Bien que la survenue d'une pachyméningite dans un contexte de SGS soit exceptionnelle, un tel syndrome doit être évoqué dans le cadre du bilan étiologique d'une pachyméningite.

**Figure 1:**
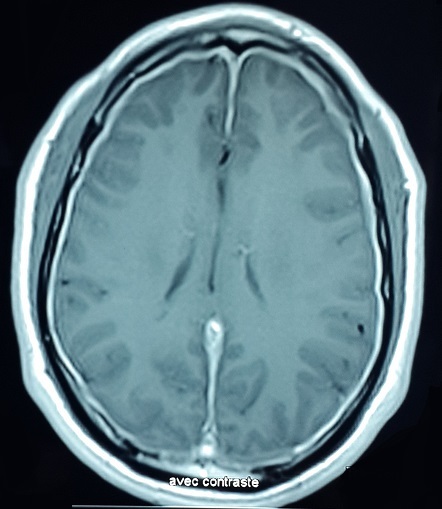
IRM cérébrale: prise de contraste intense et épaissie des espaces méningés sous et sus-tentoriels réalisant l'aspect d'une pachyméningite diffuse

